# Efficacy and safety of ChondroT on knee-osteoarthritis: Protocol for a 8-week, randomized, double-blind, placebo-controlled, multicenter therapeutic exploratory clinical trial

**DOI:** 10.1097/MD.0000000000010170

**Published:** 2018-03-23

**Authors:** Sangkwan Lee, Seon-jong Kim

**Affiliations:** a College of Korean Medicine, Wonkwang University, Iksandae-ro, Iksan, Jeonbuk, Republic of Korea; b College of Korean Medicine, Dongshin University, Geonjae-ro, Naju, Jeollanam-do, Republic of Korea.

**Keywords:** complementary therapy, osteoarthritis, randomized double-blind placebo-controlled trials

## Abstract

**Background:**

Arthritis is the most common disease in elderly individuals. Many medications for osteoarthritis treatment have the potential for side effects. Using a bioinformatics tool and preclinical studies, ChondroT, 5 herbal complexes, was identified from Ganghwaljetongyeum, which is a18 herbal complex, which has often used to treat osteoarthritis. The goal of this study is to evaluate short-term safety of ChondroT.

**Methods:**

This will be a multicenter, randomized, double-blind, and placebo-controlled trial. There will be a 2-week run-in period before random allocation to 3 groups, ChondroT 1.0 g, 2.0 g, and placebo groups. Total duration of the clinical trial will be 14 weeks including a 4-week washout follow-up. Participants will be followed-up every 4 weeks, and the effect and the safety will be assessed at visit 2, 3, and 4. All participants are asked to maintain the medication schedule in this protocol. The primary outcome will be measured using pain visual analog scale (VAS) after 8 week treatment and secondary outcomes will include pain VAS score after 4 week treatment, SF-36 survey score, patient's global assessment, physical function test, and the change of erythrocyte sedimentation rate, and C-reactive protein. The repeated-measure analysis will be used for the primary efficacy based on full analysis set and per-protocol.

**Discussion:**

This study has restrictive inclusion, exclusion criteria, and a well-controlled intervention, and it will be the first randomized controlled trial to evaluate the efficacy and safety of ChondroT formula in osteoarthritis patients. The trial according to this protocol may provide a new intervention in the treatment of osteoarthritis.

## 1 Introduction

Arthritis is the most common inflammatory disease and a major public concern in elderly individuals. The symptoms are characterized by inflammation, pain, and stiffness in the musculoskeletal system and they range from localized self-limiting conditions to systemic autoimmune processes.^[1]^

Various modalities to manage knee joint pain range from conservative management, such as exercise, oral medication including nonsteroidal antiinflammatory agents, and joint injection, to surgical treatment. All medications for osteoarthritis have the potential for side effects^[2–4]^ depending on the type of drugs or individuals involved.

Ganghwaljetongyeum (GHJTY), a mixture of traditional herbs, also has been widely used to improve the symptoms of osteoarthritis in China and Korea. Our previous study showed that GHJTY was effective on rheumatoid arthritis by inhibiting the production of pro-inflammatory mediators and proliferation of synoviocytes.^[5]^ It is a complex herbal decoction composed of 18 plants. In order to improve the efficacy and convenience of pharmaceutical prescription, a previous bioinformatics study^[6]^ identified the 5 medicinal herbs with the greatest potential. These were *Ostericum koreanum* Maximowicz (Osterici Radix, OK), *Lonicera japonica* Thunberg (Lonicerae Folium, LJ), *Clematis mandshurica* Ruprecht (Clematis Radix, CM), *Angelica gigas* Nakai (AngelicaeGigantis Radix, AG), and *Phellodendron amurense* Ruprecht (Phellodendri Cortex, PA). The 5 herbs were extracted with water and named ChondroT. A previous study confirmed that ChondroT had a chondroprotective effect and demonstrated multitarget mechanisms related to inflammation and arthritis which suggest ChondroT's therapeutic potential for the treatment of arthritis.^[7]^

In this study, we designed this study protocol to test if this new drug, ChondroT, is effective on symptoms of osteoarthritis patients by randomized, double-blind, placebo-controlled, multicenter clinical trials.

## 2 Methods and design

### 2.1 Study design

**Figure 1:**
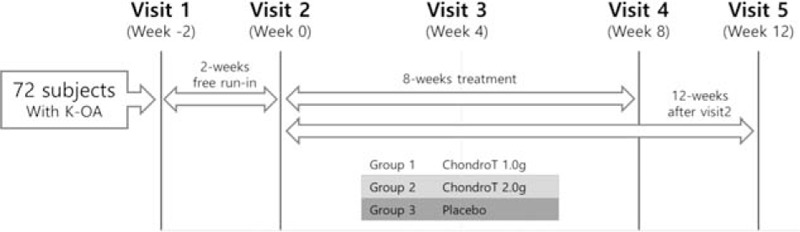
Study design for ChondroT clinical trial. There is a 2-week run-in period before randomization, and follow-up will be 12 weeks including 8-week treatment period with visit once every 4 weeks, and 4-week washout period.

This will be a multicenter, randomized, double-blind, and placebo-controlled trial. Participants will be recruited through poster or advertisement from arthritis outpatient department in Dongshin University Korean medicine hospitals in Gwangju or Mokpo (DSGOH or DSMPOH). There will be a 2-week run-in period before random allocation to 3 groups, ChondroT 1.0 g, 2.0 g and placebo groups. Total duration of the clinical trial will be 14 weeks including a 4-week washout follow-up. Participants will be followed-up every 4 weeks, and the effect and the safety will be assessed at visit 2, 3, and 4 (Fig. 1).

### 2.2 Ethics approval and consent to participate

The protocol (version 4.51) of this study has been approved by ethical approval from the institutional review board (IRB) in DSGOH (DSGOH-044, 2017.07.17) and DSMPOH (DSMPOH 17-1, 2017.07.05). For those eligible patients, researchers will give participants the detailed information of this study. Participants or their family should give written informed consent, and then will be enrolled in the study.

Subjects may be required to quit the study in case of serious adverse events or adverse drug reaction, and then they will be reported to institutional review board in both hospitals. In addition, patients may leave this study at any time without any disadvantage or constraint.

Trials are being conducted by supervision of the clinical trial center in both hospitals and monitored by an independent contract research organization (CRO). Especially, a specific clinical research coordinator will coordinate all procedures for participants to improve participants’ adherence to intervention protocols. If there is any change in protocol, it will be approved by IRB again before implementation.

The results will be published on the website of ClinicalTrials.gov in accordance with the CONSORT 2010 Statement and in special journals.

### 2.3 Selection and inclusion criteria

Inclusion criteria were as follows: diagnosed as knee osteoarthritis according to the clinical diagnosis of American College of Rheumatology clinical classification criteria;^[8]^ a daily mean pain visual analog scale (VAS) (100 mm Pain VAS) ≥ 40, during the past 1 week (0–100); male or female aged 50 to 80 years.

Exclusion criteria were as follows: receiving immunosuppressant, corticosteroids, cyclosporine, antipsychotics, osteoarthritis medications including painkillers (pas, spray, etc.) or health functional foods to improve joint or cartilage health; having renal functional disorder (more than twice the normal limit of serum creatinine), liver functional disorder (exceeds the normal limit by more than 3 times), connective tissue, multiple sclerosis, inflammatory disease, tumor, trauma, rheumatoid arthritis, autoimmune disease, severe genu valgum or vagus, secondary arthritis, hip disease, or other forms of inflammatory arthritis disease; having a past history such as knee surgery including arthrocentesis drainage or gastrointestinal operations (except for simple appendicitis or hernia) or abuse of drug or alcohol; being treated with traditional medical interventions, steroids, or mucosal supplements injection (hylane, sodium hyaluronate, hyalulonan, etc.) in the knee joint or physical therapy; participating in other clinical trials within 2 months before the screening.

### 2.4 Intervention and control

All participants will take 2-week run-in period before randomization, then receive a treatment of 3 groups, ChondroT 1.0 g (2 chondroT tablets + 2 placebo tablets), ChondroT 2.0 g (4 chondroT tablets) or placebo tablet (Table 1) in the following 8-week treatment period. Drugs will be manufactured by Jeongwoo Pharmaceutical Co. and 2 tablets will be orally administered twice daily.

**Table 1:**
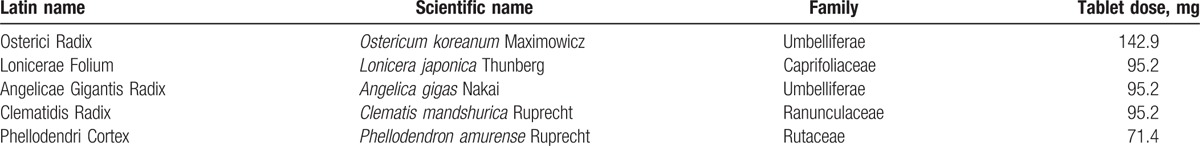
Composition of ChondroT tablet.

### 2.5 Randomization

Patients will be assigned a patient number, checked whether they meet all the criteria for the enrolment, and then randomized to double blind treatment in a 1:1:1 ratio according to the sequence generated by a computer random program. Both researchers and participants will not know the assignment group because of mimic appearance, smell and taste between ChondroT and placebo treatment drugs. The information on intervention assignment will be stored in the third department of statistics.

### 2.6 Outcomes

The primary outcome is pain VAS score, a self-reporting scale for rating severity of pain in osteoarthritis, after 8 week treatment. The secondary outcomes are pain VAS score after 4 week treatment, SF-36 survey score^[9]^ for rating quality of life, patient's global assessment for rating satisfaction of treatment,^[10]^ physical function test for rating functional improvement, and the change of erythrocyte sedimentation rate and C-reactive protein representing inflammation in the body. For safety of drugs, we will conduct the laboratory test, ECG, and vital signs. Safety assessments will be conducted every visit. The assessment of outcomes will be performed by 2 blinded doctors and the average of the 2 doctors’ scores will be determined as final scores.

Participants’ data will be anonymized and coded by a specific program. Double data entry will be entered and monitored by an expert in independent CRO to promote data quality. The final data will be assessed by the principal investigator and the independent statistician. All data during the trial will be stored in archive at clinical trial center in both hospitals.

### 2.7 Statistical analysis

Full analysis set (FAS) and per-protocol set (PPS) will be used for the efficacy assessment. According to the principle of intentionality treatment, all patients having the efficacy assessment record at least one time will be brought into the FAS. PPS will include patients who complete the trial and do not violate the protocol.

The primary goal of this study is to test whether ChondroT, a herbal complex, is effective on osteoarthritis patients. Repeated-measures analyses will be used to assess between-group differences in the modeled change in scores from baseline to 12 weeks. Independent variables for fixed effects will be treatment (3 levels: ChondroT 1.0 g, ChondroT 2.0 g and placebo), visit (4 levels: baseline, week 4, week 8, and week 12), interaction between treatment and visit, baseline measures, sex, age, and so on.

For safety analysis, Chi-square test, Fisher exact test, or Wilcoxon rank test will be conducted for assessing the differences of incidence of adverse events (symptoms, signs, diseases, laboratory tests, or abnormal electrocardiogram manifestations) between ChondroT and placebo groups.

All analyses will be performed using the software of Statistics Analysis System, blinded to treatment allocation.

### 2.8 Sample size

Considering the phase of this study (2a) to evaluate short-term safety of a drug, we have not calculated the sample size based on the previous studies. The sample size is 72 in total and 24 for each group. Assuming a dropout of about 20% and sufficient statistical power of 80% at a significant level of 0.05 in 2 tailed test, about 57 patients may be expected to complete the trials finally.

## 3 Discussion

Arthritis is the most common inflammatory disease in elderly individuals. The symptoms are characterized by inflammation, pain, and stiffness mainly in the musculoskeletal system.^[1]^

Many medications for osteoarthritis have the potential for side effects.^[2–4]^ Therefore, demands for the development of safer medication have been increased. Our previous study showed that GHJTY, a 18 herbal complex, was effective on rheumatoid arthritis by inhibiting the production of pro-inflammatory mediators and proliferation of synoviocytes.^[5]^ Other previous studies^[6]^ identified that the 5 medicinal herbs had the greatest potential, named ChondroT, from GHJTY to improve the efficacy and convenience of pharmaceutical prescription using bioinformatic method and confirmed that ChondroT had a chondroprotective effect and demonstrated multitarget mechanisms related to inflammation and arthritis which suggest ChondroT's therapeutic potential for the treatment of arthritis.^[7]^

The phase of this study is trial 2a to evaluate short-term safety of the drug. It will be a study with restrictive inclusion, exclusion criteria, and a well-controlled intervention, and will be the first randomized controlled trial to evaluate the efficacy and safety of ChondroT formula in osteoarthritis patients. However, this protocol may have limitations of the small sample size which may lead to bias. The trial according to this protocol may provide a new intervention in the treatment of osteoarthritis.

## 4 Author contributions

**Conceptualization:** S. Lee.

**Writing – original draft:** S. Lee.

**Writing – review & editing:** S-J. Kim.
